# Comprehensive analysis of the expression, prognostic significance, and function of *FAM83* family members in breast cancer

**DOI:** 10.1186/s12957-022-02636-9

**Published:** 2022-06-01

**Authors:** Yi Jin, Jiahui Yu, Yi Jiang, Jiawen Bu, Tong Zhu, Xi Gu, Xudong Zhu

**Affiliations:** 1 Department of Breast Surgery, Cancer Hospital of China Medical University, Liaoning Cancer Hospital and Institute, Shenyang, Liaoning 110042 People’s Republic of China; 2 Department of Ultrasound, Shengjing Hospital of China Medical University, Shenyang, Liaoning 110004 People’s Republic of China; 3 Department of Oncology, Shengjing Hospital of China Medical University, Shenyang, Liaoning 110004 People’s Republic of China; 4 Department of General Surgery, Cancer Hospital of China Medical University, Liaoning Cancer Hospital and Institute, Shenyang, Liaoning 110042 People’s Republic of China

**Keywords:** Breast cancer, *FAM83* family, Therapeutic targets

## Abstract

**Background:**

The *FAM83* family plays a key role in tumorigenesis and cancer progression. However, the role of the *FAM83* family in the development of breast tumors is unclear to date. This report explores the expression, prognostic significance, and function of the *FAM83* family members in breast cancer using public databases.

**Methods:**

UALCAN database was used to explore the expression of *FAM83* family members in breast cancer. Furthermore, we validated the expression of *FAM83* family members in twenty pairs of breast cancer and normal tissues by RT-PCR. Kaplan–Meier plotter database was used to explore the prognostic significance of *FAM83* family members in breast cancer. GeneMANIA and DAVID databases were used for functional and pathway enrichment analysis of genes co-expressed with FAM83A, FAM83D, FAM83F, and FAM83G. MEXPRESS and UALCAN databases were used to analyze the level of DNA promoter methylation of FAM83A, FAM83D, FAM83F, and FAM83G in breast cancer. TIMER database was utilized to explore the relationships between immune cell infiltration and FAM83A, FAM83D, FAM83F, and FAM83G expression.

**Results:**

Among *FAM83* family members, FAM83A, FAM83D, FAM83F, and FAM83G were higher expressed in breast cancer than in normal tissues. We also validated the significant high expression of FAM83A, FAM83D, FAM83F, and FAM83G mRNA in breast cancer than in normal samples. Their increased expression has an adverse prognostic effect on breast cancer patients. These genes co-expressed with FAM83A, FAM83D, FAM83F, and FAM83G might take part in cell proliferation, G2/M transition of the mitotic cell cycle, regulation of apoptosis process and other cancer-related biological processes. In addition, they were mainly enriched in the Hippo signaling pathway, Hedgehog signaling pathway, PI3K/AKT signaling pathway, and other cancer-related pathways. We also found that promoter DNA methylation might regulate the expression of FAM83A, FAM83D, FAM83F, and FAM83G mRNA in most CpG islands. At last, we found the expression of FAM83A, FAM83D, FAM83F, and FAM83G mRNA was significantly related to immune cell infiltration.

**Conclusions:**

FAM83A, FAM83D, FAM83F, and FAM83G were highly expressed in breast cancer tissues and had an adverse effect on the survival outcomes of breast cancer patients. Also, they were involved in breast cancer-related signal pathways. Therefore, they might serve as potential therapeutic targets for breast cancer clinical treatment.

**Supplementary Information:**

The online version contains supplementary material available at 10.1186/s12957-022-02636-9.

## Background

Breast cancer has been the most common malignancy in women around the world and the second leading cause of cancer death in women. The number of new breast cancer cases accounted for about 30% of the total number of all new malignant tumors in women every year, representing a significant threat to women’s health and wellness worldwide [1]. However, two breakthroughs were achieved in the last decades: the establishment of molecular subtypes of breast cancer and the emergence of comprehensive treatment methods [2, 3]. Early tumor detection and subtype classification, together with new treatments, have improved the survival outcomes of breast cancer patients. However, for some patients with resistant cancers receiving endocrine therapy, chemotherapy, targeted therapy, or even immunotherapy with no obvious response, it was imperative to unveil new potential targets for cancer treatment [4].

Recent studies have found that the proteins of the FAMily with sequence similarity 83 (*FAM83*) family possess oncogenic properties, and their expression levels were elevated in many human cancers [5]. To date, evolutionarily speaking, people found that not all organisms encoded the *FAM83* gene. For example, some lower organisms such as Drosophila, Saccharomyces cerevisiae and Caenorhabditis elegans. But all jawed vertebrates encoded the *FAM83* gene [6]. The FAM83 family comprised eight protein members: FAM83A-FAM83H. All the FAM83 family members shared a conserved N-terminal DUF1669 domain with unknown functions [5, 7, 8]. The DUF1669 domain consisted of a conserved phospholipase D (PLD)-like catalytic motif. But the FAM83 proteins displayed no true PLD catalytic (PLDc) activity, and the pseudo-PLDc motif that each FAM83 member had lacked the crucial elements of the native PLD catalytic motif. Owing to the absence of catalytic activity, the DUF1669 domain may have evolved to espouse novel functions in biology [9]. Outside the DUF1669 domain, there were no similar sequences among family members. All FAM83 proteins bound to casein kinase 1(CK1) family of Ser/Thr protein kinases through the DUF1669 domain and participated in the regulation of isoenzymes of CK1. FAM83 members directly controlled the subcellular localization of CK1, as well as their activity, stability and substrate specificity by binding to CK1, thereby limiting the function of CK1 in the cells [10]. Mutations in the DUF1669 domain can eliminate the interaction with CK1, thereby interfering with the FAM83 member itself and their CK1 binding partners. Owing to the members of the CK1 family were implicated in the regulation of many cellular processes, including the cell cycle, circadian rhythms, and Wnt and Hedgehog signaling [11], we think the FAM83 family members may also be involved in these processes.

The firstly identified FAM83 proteins were FAM83A and FAM83B [12, 13]. Subsequently, some studies proved that *FAM83* family proteins overexpression and dysregulation play a role in cancer cell proliferation, invasion, metastasis, and resistance to specific drug treatments [14, 15]. Although previous reports have found that some *FAM83* family members are expressed in breast tumors, there was limited in-depth research about the function of *FAM83* family proteins in breast malignancies [12, 13, 16]. Therefore, a comprehensive study of the expression patterns of the FAM83 family proteins in breast cancer can help to shed some light on the molecular mechanisms involved in breast cancer development and might unveil novel prognostic and therapeutic targets of interest for the pharmaceutical industry.

Therefore, in this report, we firstly explored the expression of the gene expression levels of the FAM83 family in breast cancer compared to normal tissues using different publicly available databases. And we validated the gene expression levels of the FAM83 family in breast cancer and healthy breast tissue samples using RT-qPCR. Then, we explored the prognostic effect of the *FAM83* family gene expression on breast cancer and identified that only some members—FAM83A, FAM83D, FAM83F, and FAM83G—were highly expressed in breast cancer and had a negative effect on the patient’s survival outcome. By analyzing their co-expressed genes, we further explored the functions and pathways of FAM83A, FAM83D, FAM83F, and FAM83G. We hope they may serve as potential therapeutic targets for breast cancer clinical treatment.

## Methods

### UALCAN analysis

The UALCAN database (ualcan.path.uab.edu/index.html) was used to analyze the mRNA levels of *FAM83* family genes in breast cancer and normal tissues. The mRNA expression level of FAM83A, FAM83D, FAM83F, and FAM83G was also analyzed in breast cancer patients based on patients’ age, individual cancer stages, menopause status, nodal metastasis status, and breast cancer subclasses. At last, we analyzed the level of FAM83A, FAM83D, FAM83F, and FAM83G promoter methylation in breast cancer and normal tissues by UALCAN database [17].

### Kaplan–Meier plotter analysis

The Kaplan–Meier plotter (http://kmplot.com; mRNA gene chip KM Plotter for breast cancer) was used to analyze the effect of the *FAM83* family gene expression on relapse-free survival (RFS) and overall survival (OS) at RNA-sequence level [18].

### The Human Protein Atlas (HPA) database

HAP database (www.proteinatlas.org) was made available freely to provide the expression profiles at protein levels, and immunohistochemistry images for a wide variety of cancer tissues. Genome‐wide transcriptomics data and clinical metadata of almost 8000 patients were also used to analyze the proteome of 17 major cancer types including breast cancer [19]. In our study, we also got the subcellular localization, the mRNA level of different cell lines ordered by organ of phenotypic resemblance, immunofluorescence staining result of these targeted genes.

### GeneMANIA analysis

To evaluate the functions of FAM83A, FAM83D, FAM83F, and FAM83G, the GeneMANIA database was used. GeneMANIA (http://www.genemania.org) was designed to construct an interaction network in terms of physical interactions, co-expression, predictions, and genetic interaction [20]. The DAVID database (https://david.ncifc rf.gov) was employed to conduct a functional and pathway enrichment analysis of the genes co-expressed with FAM83A, FAM83D, FAM83F, and FAM83G [21].

### MEXPRESS analysis

The MEXPRESS database (https://mexpress.be/) was employed to analyze the level of DNA promoter methylation of FAM83A, FAM83D, FAM83F, and FAM83G in breast invasive carcinoma (BRCA) [22].

### TIMER

The TIMER database (Tumor Immune Estimation Resource: https://cistrome.shinyapps.io/timer/), a user-friendly web-based interface, was used to systematically evaluate the tumor infiltration of different immune cells [23]. FAM83A, FAM83D, FAM83F, and FAM83G genes were selected, and their expression was plotted against immune cell infiltration levels in breast invasive carcinoma. We further validated the relationships between their expression and StromalScore, ImmuneScore, and ESTIMATEscore.

### The Comparative Toxicogenomics Database

The Comparative Toxicogenomics Database (CTD) was an innovative online database that provided literature-based information on the interactions between oncogene products and chemotherapeutic compounds. We used this tool to screen potential therapeutic compounds that could target and reduce the mRNA expression of FAM83A, FAM83D, FAM83F, and FAM83G [24].

### RT-qPCR

Total RNA extracted from twenty pairs of fresh breast cancer and healthy breast tissues were isolated using Trizol solution (Solarbio; R1100). Extracted RNA was then reverse-transcribed using a cDNA synthesis kit (TaKaRa) following the manufacturer’s instructions. qPCR was performed using the SYBR Green PCR Master Mix (TaKaRa) and primers binding to the *FAM83* family genes and *GAPDH* gene on the Fast-Real-Time PCR System. *GAPDH* was used as a reference gene. The primers were designed by Shanghai Sangon Biotech Co., Ltd., and the sequence of primers can be found in Supplementary Table 1. The cycling protocol used was as follows: 95 °C for 30 s (initial denaturation), followed by 40 cycles of 95 °C for 3 s (denaturation), and 60 °C for 30 s (annealing and extension). The relative level of mRNA was calculated using the 2^−ΔΔCt^ method [25]. These breast cancer specimens were obtained from China Medical University at the time of surgery, and the basic clinicopathological characteristics of these twenty patients were shown in Supplementary Table 2. This study was approved by the Institutional Review Board of China Medical University.

### Statistical analysis

The comparison between the expression level of FAM83 family mRNAs in cancer tissues and normal tissues were calculated by independent sample *t* test. All these *P* values were two-sided, and the significance was at *P* < 0.05. Unless otherwise stated, analyses were performed by SPSS 25.0 software.

## Results

### Exploration of the gene expression levels of the FAM83 family in breast cancer and normal tissues using the UALCAN database

**Fig. 1:**
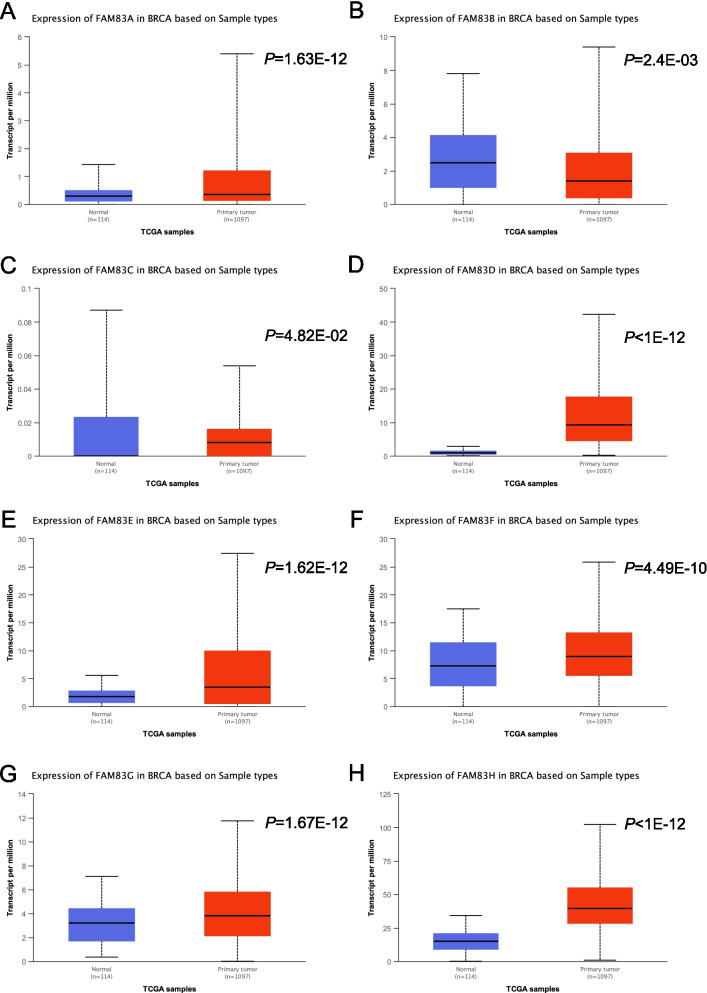
Gene expression levels of the *FAM83* family genes in breast cancer and normal breast tissues by UALCAN database. Expression of **A** FAM83A, **B** FAM83B, **C** FAM83C, **D** FAM83D, **E** FAM83E, **F** FAM83F, **G** FAM83G, and **H** FAM83H in breast cancer based on sample types. (The solid line in the middle represented the mean value of gene expression, and the upper and lower solid lines represented the mean value plus or minus standard deviation of gene expression. The dotted line connected the mean and standard deviation in every picture. The solid lines and dotted lines in these pictures obtained from UALCAN database meant the same in this paper)

Firstly, we explored the expression of *FAM83* family genes using the UALCAN database (Fig. 1). We found that the expression of FAM83A, FAM83D, FAM83E, FAM83F, FAM83G, and FAM83H were significantly higher in breast cancer tissues compared to normal tissues (*P* = 1.63E − 12, Fig. 1A; *P* < 1E − 12, Fig. 1D; *P* = 1.62E − 12, Fig. 1E; *P* = 4.49E − 10, Fig. 1F; *P* = 1.67E − 12, Fig. 1G; *P* < 1E − 12, Fig. 1H). However, the expression of FAM83B was significantly lower in breast cancer tissues compared to normal tissues (*P* = 2.4E − 03, Fig. 1B), and no significant differences were found in FAM83C expression levels (*P* = 4.8E − 02, Fig. 1C).

### Validation of the mRNA expression of FAM83 family genes in fresh breast cancer and normal tissues samples

**Fig. 2:**
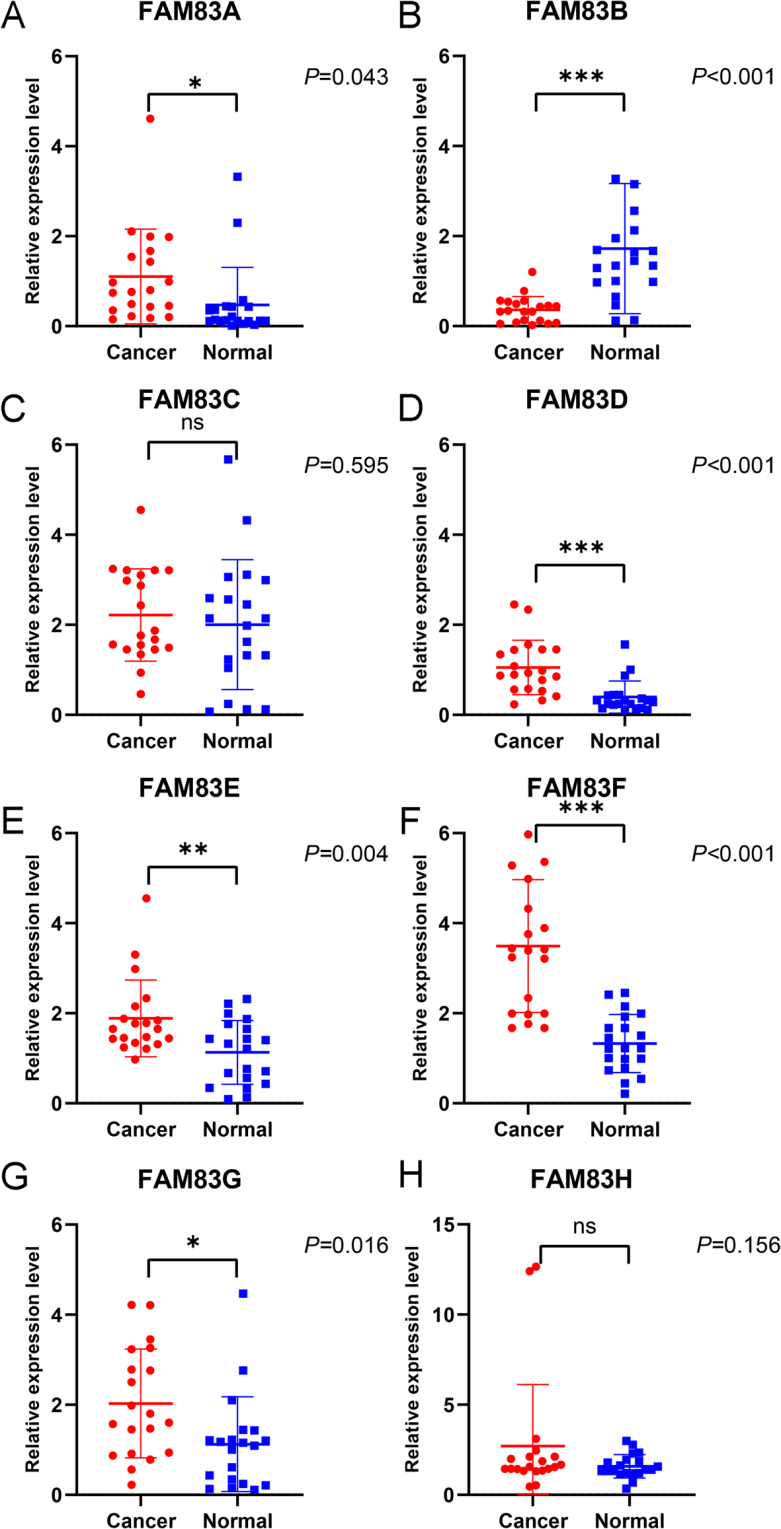
Validation of the mRNA expression of *FAM83* family genes in fresh breast cancer and healthy breast tissue samples. Validation of **A** FAM83A, **B** FAM83B, **C** FAM83C, **D** FAM83D, **E** FAM83E, **F** FAM83F, **G** FAM83G, **H** FAM83H expression by RT-PCR. (The solid line in the middle represented the mean value of gene expression, and the upper and lower solid lines represented the mean value plus or minus standard deviation of gene expression. The vertical solid line connected the mean and standard deviation in every picture)

Next, we validated the mRNA expression of *FAM83* family genes in human tissues using 20 pairs of breast cancer and healthy tissues. We found that the expression of FAM83B was significantly lower in breast cancer tissues than in normal tissues (*P* < 0.001, Fig. 2B). On the other hand, the expression of FAM83A, FAM83D, FAM83E, FAM83F, and FAM83G was significantly higher in breast cancer tissues than in normal tissues (*P* = 0.043, Fig. 2A; *P* < 0.001, Fig. 2D; *P* = 0.004, Fig. 2E; *P* < 0.001, Fig. 2F; *P* = 0.016, Fig. 2G). No significant differences were found for FAM83C and FAM83H (*P* = 0.595, Fig. 2C; *P* = 0.156, Fig. 2H).

### Exploration of the effect of the gene expression levels of the FAM83 family on relapse-free survival of breast cancer patients

**Fig. 3:**
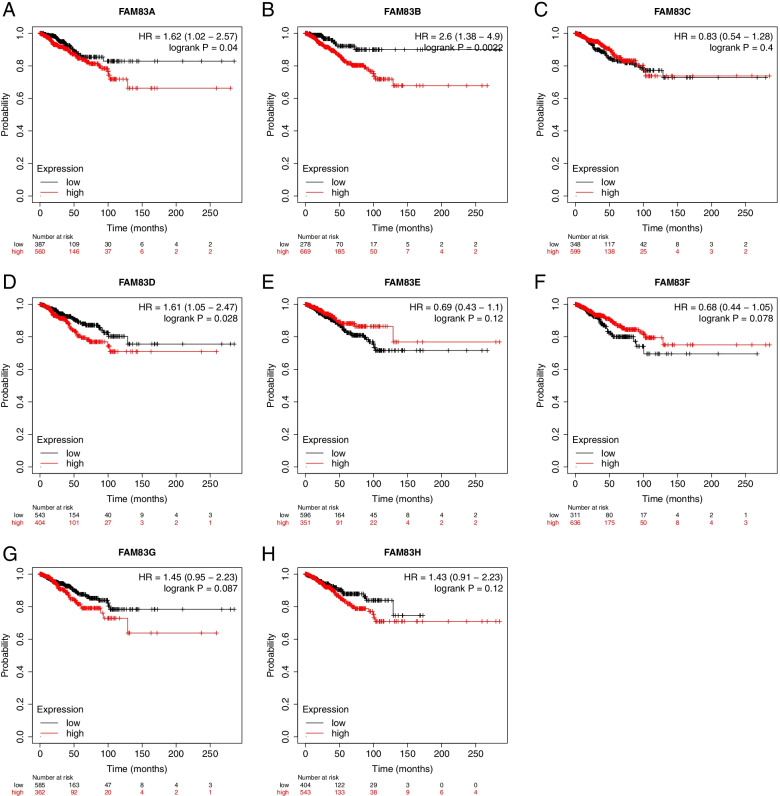
The effect of the gene expression levels of the *FAM83* family on the RFS of breast cancer patients. Effect of **A** FAM83A, **B** FAM83B, **C** FAM83C, **D** FAM83D, **E** FAM83E, **F** FAM83F, **G** FAM83G, and **H** FAM83H expression on the RFS of breast cancer patients

Next, we explored the effect of the *FAM83* family gene expression on relapse-free survival (RFS). We found that not all members of the FAM83 family had the same effects on RFS. High expression of FAM83A (hazard ratios (HR) = 1.62 (1.02–2.57); *P* = 0.04; Fig. 3A), FAM83B (HR = 2.6 (1.38–4.9); *P* = 0.0022; Fig. 3B), and FAM83D (HR = 1.61 (1.05–2.47); *P* = 0.028; Fig. 3D) were found to be significantly related to shorter RFS. However, high expression of FAM83C (HR = 0.83 (0.54–1.28); *P* = 0.4; Fig. 3C), FAM83E (HR = 0.69 (0.43–1.1); *P* = 0.12; Fig. 3E), FAM83F (HR = 0.68 (0.44–1.05); *P* = 0.078; Fig. 3F), FAM83G (HR = 1.45 (0.95–2.23); *P* = 0.087; Fig. 3G), and FAM83H (HR = 1.45 (0.91–2.23); *P* = 0.12; Fig. 3H) were not related to shorter RFS. Therefore, for RFS, only three of the eight family members—FAM83A, FAM83B, and FAM83D—had an adverse effect on patients’ survival.

### Exploration of the effect of the gene expression levels of the FAM83 family on overall survival of breast cancer patients

**Fig. 4:**
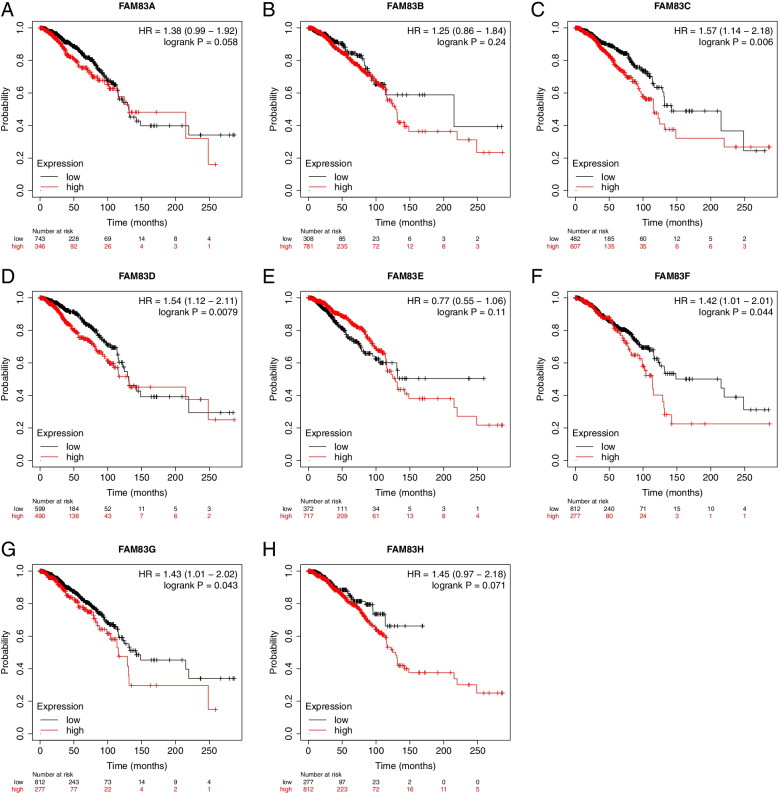
The effect of the gene expression levels of the *FAM83* family on the OS of breast cancer patients. Effect of **A** FAM83A, **B** FAM83B, **C** FAM83C, **D** FAM83D, **E** FAM83E, **F** FAM83F, **G** FAM83G, and **H** FAM83H expression on the OS of breast cancer patients

Next, we investigated the effect of the gene expression levels of the *FAM83* family on overall survival (OS). Similar to RFS, we found that not all members of the FAM83 family had the same effect on OS. High expression of FAM83C (HR = 1.57 (1.14–2.18); *P* = 0.006; Fig. 4C), FAM83D (HR = 1.54 (1.12–2.11); Fig. 4D), FAM83F (HR = 1.42 (1.01–2.01); *P* = 0.044; Fig. 4F), and FAM83G (HR = 1.43 (1.01–2.02); *P* = 0.043; Fig. 4G) were significantly related to a shorter OS. On the other hand, FAM83A (HR = 1.38 (0.99–1.92); *P* = 0.058; Fig. 4A), FAM83B (HR = 1.25 (0.86–1.84); *P* = 0.24; Fig. 4B), FAM83E (HR = 0.77 (0.55–1.06); *P* = 0.11; Fig. 4E), and FAM83H (HR = 1.45 (0.97–2.18); *P* = 0.071; Fig. 4H) were not significantly related to a shorter OS. These results indicate that, for OS, four of the eight members of the FAM83 family—FAM83C, FAM83D, FAM83F, and FAM83G—had an adverse effect on patients’ overall survival.

Based on the analysis above, the following genes were selected for downstream analysis: FAM83A, FAM83D, FAM83F, and FAM83G. The oncogene selection was based on two criteria: high gene expression in breast cancer tissues and adverse effects on patients’ RFS or OS.

### Exploration of the gene expression of FAM83A, FAM83D, FAM83F, and FAM83G based on different clinicopathological characteristics by UALCAN database

The UALCAN database was a powerful integrated data-mining platform for analyzing cancer OMICS data. Hence, we utilized this database to explore the expression levels of the selected oncogenes based on patients’ age, individual cancer stage, menopause status, nodal metastasis stats, and breast cancer subclasses.

For FAM83A, 21–40 years old patients have the highest FAM83A expression (*P* = 8.22E − 04). However, among breast cancer patients in different age subgroup, the difference in FAM83A expression was not significant (Supplementary Fig. 1A). Stage 2 patients have the highest FAM83A expression (*P* = 4.51E − 10). Among breast cancer patients in different subgroup of individual cancer stage, the expression of FAM83A in stage 2 patients was significantly higher than in stage 1 patients, the difference in other comparison was not significant (Supplementary Fig. 1B). Pre-menopause patients have the highest FAM83A expression (*P* = 1.42E − 06). However, among breast cancer patients in different subgroup of menopause status, the difference in FAM83A expression was not significant (Supplementary Fig. 1C). N3 patients have the highest FAM83A expression (*P* = 7.36E − 04). Among breast cancer patients in different subgroup of nodal metastasis status, the expression of FAM83A in N3 patients was significantly higher than in N1 patients, the difference in other comparison was not significant (Supplementary Fig. 1D). At last, HER2-positive patients have the highest FAM83A expression (*P* = 1.98E − 02). The expression of FAM83A in HER2-positive patients was also significantly higher than in luminal patients and triple negative breast cancer (TNBC) patients (Supplementary Fig. 1E).

For FAM83D, 21–40 years old patients have the highest FAM83D expression (*P* = 1.63E − 12). The expression of FAM83D in 21–40 years old patients was significantly higher than in 61–80 years old patients. The expression of FAM83D in 21–40 years old patients was also significantly higher than in 81–100 years old patients. Additionally, the expression of FAM83D in 41–60 years old patients was significantly higher than in 81–100 years old patients. The difference in other comparison was not significant (Supplementary Fig. 2A). Stage 4 patients have the highest FAM83D expression (*P* = 3.42E − 06). Among breast cancer patients in different subgroup of individual cancer stage, both the expression of FAM83D in stage 2 and stage 3 patients were significantly higher than in patients with stage 1. The expression of FAM83D in stage 4 patients was significantly higher in patients with stage 2. The difference in other comparison was not significant (Supplementary Fig. 2B). Peri-menopause patients have the highest FAM83D expression (*P* = 1.62E − 12). However, among breast cancer patients in different subgroup of menopause status, the difference in FAM83D expression was not significant (Supplementary Fig. 2C). N2 patients have the highest FAM83D expression (*P* = 1.62E − 12). Among breast cancer patients in different subgroup of nodal metastasis status, the difference in all comparison was significant except in “N0 vs N1” and “N0 vs N2”. The result was shown in Supplementary Fig. 2D. At last, TNBC patients have the highest FAM83D expression (*P* = 1.62E − 12). And among breast cancer patients in different subgroup of major subclasses, the differences in all comparison were significant. The result was shown in Supplementary Fig. 2E.

For FAM83F, 21–40 years old patients have the highest FAM83F expression (*P* = 2.02E − 05). However, among breast cancer patients in different age subgroup, the difference in FAM83F expression was not significant (Supplementary Fig. 3A). Stage 4 patients have the highest FAM83F expression (*P* = 4.78E − 02). Among breast cancer patients in different subgroup of individual cancer stage, the difference in FAM83F expression was not significant (Supplementary Fig. 3B). Post-menopause patients have the highest FAM83F expression (*P* = 2.80E − 07). Among breast cancer patients in different subgroup of menopause status, the difference in FAM83F expression was not significant (Supplementary Fig. 3C). N3 patients have the highest FAM83F expression (*P* = 6.44E − 03). Among breast cancer patients in different subgroup of nodal metastasis status, the difference in all comparison was not significant except in “N0 vs N1” and “N0 vs N3”. The result was shown in Supplementary Fig. 3D. At last, TNBC patients have the highest FAM83F expression (*P* = 1.09E − 09). And the expression of FAM83F in TNBC patients was significantly higher than in Luminal and HER2-positive breast cancer patients. The result was shown in Supplementary Fig. 3E.

For FAM83G, 61–80 years old patients have the highest FAM83G expression (*P* = 1.93E − 11). However, among breast cancer patients in different age subgroup, the difference in FAM83G expression was not significant (Supplementary Fig. 4A). Stage 3 patients have the highest FAM83G expression (*P* = 4.21E − 07). Among breast cancer patients in different subgroup of individual cancer stage, the difference in FAM83F expression was not significant (Supplementary Fig. 4B). Post-menopause patients have the highest FAM83G expression (*P* = 2.11E − 08). The expression of FAM83G was significantly higher in pre-menopause patients than in peri-menopause patients. And the expression of FAM83G was also significantly higher in post-menopause patients than in peri-menopause patients. The result was shown in Supplementary Fig. 4C. N3 patients have the highest FAM83G expression (*P* = 1.90E − 03). Among breast cancer patients in different subgroup of nodal metastasis status, the difference in all comparison was not significant. The result was shown in Supplementary Fig. 4D. At last, TNBC patients have the highest FAM83G expression (*P* = 3.91E − 10). The expression of FAM83G in TNBC patients was significantly higher in Luminal and HER2-positive breast cancer patients. In addition, the expression of FAM83G in Luminal patients was also significantly higher than in HER2-positive breast cancer patients. The result was shown in Supplementary Fig. 4E.

### Exploration the expression and subcellular localization of FAM83A, FAM83D, FAM83F, and FAM83G by the HPA database

By the HPA database, we further explored the expression, subcellular location and other related results of FAM83A, FAM83D, FAM83F, and FAM83G. For FAM83A, we got its subcellular localization (mainly enriched in nucleoplasm and cytosol), the *FAM83A* mRNA level of different cell lines ordered by organ of phenotypic resemblance, and the immunofluorescence staining result of FAM83A in RT4 cells. These results were shown in Supplementary Fig. 5. For FAM83D, we got its subcellular localization (mainly enriched in mitotic spindle, microtubules, cytokinetic bridge and cytosol), the *FAM83D* mRNA level of different cell lines ordered by organ of phenotypic resemblance, the immunofluorescence staining result of FAM83D in A-431 cells, and the *FAM83D* mRNA expression levels in different cell cycles. These results were shown in Supplementary Fig. 6. For FAM83F, we got the typical immunohistochemical positive pictures of FAM83F in breast cancer patients’ specimens, its subcellular location (mainly enriched in mitochondria and nucleoplasm), the *FAM83F* mRNA level of different cell lines ordered by organ of phenotypic resemblance, immunofluorescence staining result of FAM83F in U-2 OS cells. These results were shown in Supplementary Fig. 7. For FAM83G, we got the typical immunohistochemical positive pictures of FAM83G in breast cancer patients’ specimens, its subcellular location (mainly enriched in cytosol), the *FAM83G* mRNA level of different cell lines ordered by organ of phenotypic resemblance, immunofluorescence staining result of FAM83G in U-251 MG cells. These results were shown in Supplementary Fig. 8.

### Functional and pathway enrichment analysis of genes co-expressed with FAM83A, FAM83D, FAM83F, and FAM83G

**Fig. 5:**
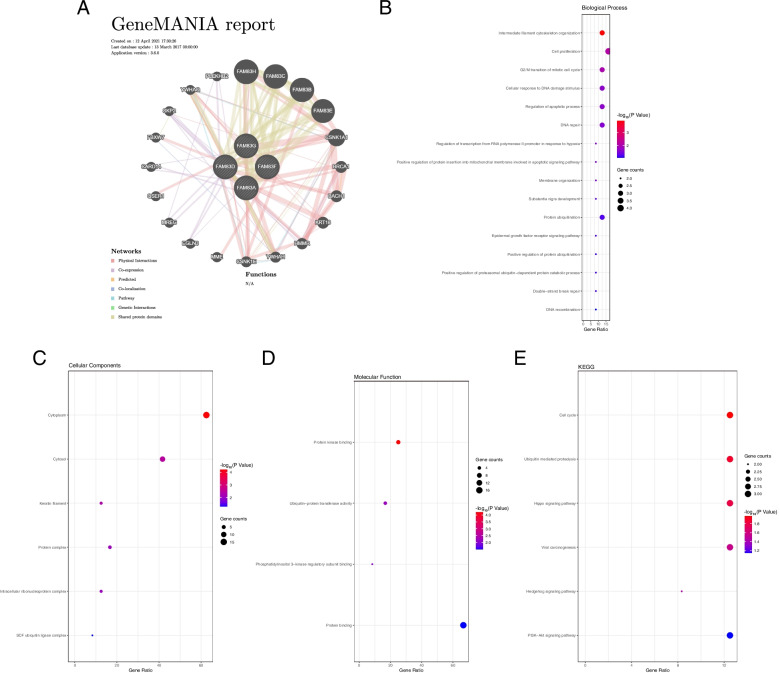
Functional and pathway enrichment analysis of genes co-expressed with FAM83A, FAM83D, FAM83F, and FAM83G. **A** The genes co-expressed with FAM83A, FAM83D, FAM83F, and FAM83G. **B** Biological process of GO functional enrichment analysis. **C** Cellular components of GO functional enrichment analysis. **D** Molecular function of GO functional enrichment analysis. **E** KEGG pathway enrichment analysis of genes co-expressed with FAM83A, FAM83D, FAM83F, and FAM83G

In order to obtain mechanistic insights, we firstly performed a protein–protein interaction analysis. We found several genes co-expressed with FAM83A, FAM83D, FAM83F, and FAM83G, including BRCA1, SKP2, and PLEKHB2 (Fig. 5A). To gain more information, we next performed a gene ontology (GO) functional and KEGG pathway enrichment analysis. In the GO analysis of biological processes, we found that these genes may take part in cell proliferation, G2/M transition of the mitotic cell cycle, regulation of apoptosis, and other biological processes depicted in Fig. 5B. In the GO analysis of cellular components, the studied genes were mainly found to be located in the cytoplasm/cytosol, protein complexes, and other locations, as shown in Fig. 5C. In GO analysis of the molecular function, we found that the genes were mainly enriched in protein kinase binding, ubiquitin-protein transferase activity, phosphatidylinositol 3-kinase regulatory subunit binding, and protein binding (Fig. 5D). The KEGG enrichment analysis indicated that these genes might take part in the Hippo, Hedgehog, and PI3K/AKT signaling pathways, among others (Fig. 5E). Taken together, our results indicated that FAM83 genes might play an essential role in the progression of breast cancer.

### Promoter DNA methylation might regulate the expression of FAM83A, FAM83D, FAM83F, and FAM83G mRNA in CpG islands

Next, we assessed the role of methylation in FAM83 gene expression. Using MEXPRESS and UALCAN analysis, we explored if the expression of FAM83A, FAM83D, FAM83F, and FAM83G mRNA was regulated by promoter DNA methylation.

**Fig. 6:**
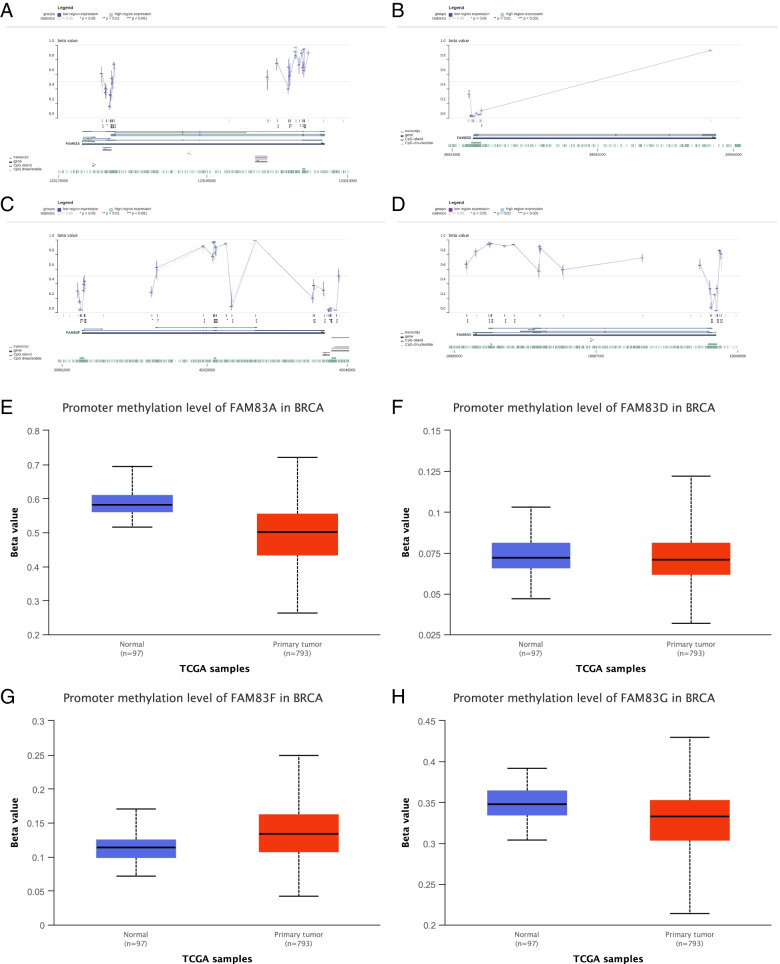
Promoter DNA methylation might regulate the expression of FAM83A, FAM83D, FAM83F, and FAM83G mRNA in most CpG islands. **A**–**D** MEXPRESS analysis. **E**–**H** UALCAN analysis

Interestingly, the tools gave slightly different results. Using MEXPRESS, we found that a low expression region was significantly associated with a high promoter DNA methylation status in most CpG islands for FAM83A, FAM83D, FAM83F, and FAM83G (Fig. 6A–D). However, in the overall analysis using the UALCAN database, we found that the level of DNA promoter methylation was significantly higher in normal tissues than in primary breast cancer tissues for FAM83A, FAM83D, and FAM83G, but not for FAM83F (Fig. 6E–H). Owing to the level of mRNA expression was significantly higher in primary breast cancer tissues than in normal tissues for FAM83A, FAM83D, and FAM83G (Fig. 1A, D, and G); therefore, we can conclude that low expression of FAM83A, FAM83D, and FAM83G, but not FAM83F, mRNA was significantly related to a high DNA promoter methylation status. This result may owe to there was not a significantly negative correlation between FAM83F mRNA expression and DNA promoter methylation in the overall analysis.

### The relationships between immune cell infiltration and FAM83A, FAM83D, FAM83F, and FAM83G expression

**Fig. 7:**
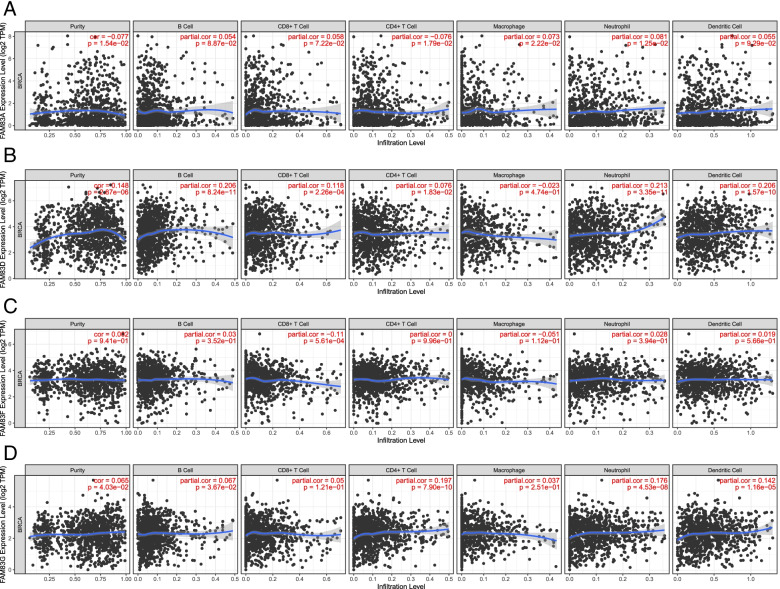
The relationships between immune cell infiltration and **A** FAM83A, **B** FAM83D, **C** FAM83F, and **D** FAM83G expression by “TIMER” analysis tool

We further explored the relationships between immune cell infiltration and FAM83A, FAM83D, FAM83F, and FAM83G expression using the “TIMER” analysis tool. The expression of FAM83A was negatively correlated with the infiltration of CD4 + T cells but positively correlated with the infiltration of macrophages and neutrophils (Fig. 7A). The expression of FAM83D was positively correlated with the infiltration of B cells, CD8 + T cells, CD4 + T cells, neutrophils, and dendritic cells (Fig. 7B). The expression of FAM83F was only found to be negatively correlated with the infiltration of CD8 + T cells (Fig. 7C). The expression of FAM83G was positively correlated with the infiltration of B cells, CD4 + T cells, neutrophils, and dendritic cells (Fig. 7D).

Besides the “TIMER” analysis tool, we also used the “ESTIMATE” analysis tool to validate these data with immune cells. As a result, we found that, for StormalScore, FAM83A expression was not significantly related to StromalScore (*P* = 0.45, Supplementary Fig. 9A). However, the expression of FAM83D, FAM83F and FAM83G was significantly negatively related to StromalScore (*P* = 3.3E − 13, Supplementary Fig. 9B; *P* = 1.9E − 8, Supplementary Fig. 9C; *P* = 8.1E − 22, Supplementary Fig. 9D). For ImmuneScore, the expression of FAM83A and FAM83D was not significantly associated with ImmuneScore (*P* = 0.12, Supplementary Fig. 9E; *P* = 0.82, Supplementary Fig. 9F). However, the expression of FAM83F and FAM83G was significantly negatively associated with ImmuneScore (*P* = 0.03, Supplementary Fig. 9G; *P* = 3.4E − 10, Supplementary Fig. 9H). Finally, for ESTIMATEScore, FAM83A expression was not significantly related to ESTIMATEScore (*P* = 0.55, Supplementary Fig. 9I). The expression of FAM83D, FAM83F, and FAM83G was significantly negatively related to ESTIMATEScore (*P* = 1.6E − 4, Supplementary Fig. 9J; *P* = 3.1E − 5, Supplementary Fig. 9K; *P* = 1.7E − 18, Supplementary Fig. 9L).

### Screening potential therapeutic compounds for breast cancer

Taking advantage of the CTD database, we aimed to screen potential therapeutic compounds that could decrease the mRNA expression of the *FAM83* oncogene in breast cancer patients.

Interestingly, we found that different members of the FAM83 family are affected by different drugs, maybe suggesting a distinct action mechanism. We found that abrine, arsenite, ethinyl estradiol, and other compounds can decrease the mRNA expression of FAM83A (Table 1). For FAM83D, doxorubicin, oxaliplatin, Palbociclib, and other compounds were found to decrease its mRNA expression (Table 2). The drugs 2,2′,3′,4,4′,5-hexachlorobiphenyl and 2-amino-2-methyl-1-propanol together with other compounds can decrease the mRNA expression of FAM83F (Table 3). FAM83G mRNA expression levels can be reduced by Cisplatin, Genistein, and other compounds (Table 4).

**Table 1:** Potential therapeutic compounds that can result in decreased expression of FAM83A mRNA

Chemical name	Chemical ID	Interaction actions	Reference count	Organism count
Abrine	C496492	decreases^expression	1	1
Arsenite	C015001	decreases^expression	1	1
Bis(4-hydroxyphenyl)sulfone	C543008	decreases^expression	1	1
Bisphenol A	C006780	decreases^expression	1	1
Butyraldehyde	C018475	decreases^expression	1	1
Cadmium chloride	D019256	decreases^expression	1	1
Chloropicrin	C100187	decreases^expression	1	1
Ethinyl estradiol	D004997	decreases^expression	1	1
Monomethylarsonous acid	C406082	decreases^expression	2	1
Silicon dioxide	D012822	decreases^expression	1	1
Sodium arsenite	C017947	decreases^expression	1	1
Sulforaphane	C016766	decreases^expression	1	1

**Table 2:** Potential therapeutic compounds that can result in decreased expression of FAM83D mRNA

Chemical name	Chemical ID	Interaction actions	Reference count	Organism count
1-Methyl-4-phenylpyridinium	D015655	decreases^expression	1	1
2,2’,4,4’-tetrabromodiphenyl ether	C511295	decreases^expression	1	1
2’,3,3’,4’,5-pentachloro-4-hydroxybiphenyl	C111118	decreases^expression	1	1
7,8-Dihydro-7,8-dihydroxybenzo(a)pyrene 9,10-oxide	D015123	decreases^expression	3	1
Aflatoxin B1	D016604	decreases^expression	1	1
afuresertib	C000593263	decreases^expression	1	1
arsenite	C015001	decreases^expression	1	1
Azathioprine	D001379	decreases^expression	1	1
Benzo(a)pyrene	D001564	decreases^expression	3	1
bisphenol A	C006780	decreases^expression	3	3
Calcitriol	D002117	decreases^expression	1	1
Cisplatin	D002945	decreases^expression	1	1
Copper Sulfate	D019327	decreases^expression	1	1
cupric oxide	C030973	decreases^expression	1	1
Cuprizone	D003471	decreases^expression	1	1
Cyclosporine	D016572	decreases^expression	4	1
dorsomorphin	C516138	decreases^expression	1	1
Doxorubicin	D004317	decreases^expression	1	1
Erucylphospho-N,N,N-trimethylpropylammonium	C472787	decreases^expression	1	1
Estradiol	D004958	decreases^expression	1	1
Estradiol 3-benzoate	C074283	decreases^expression	1	1
Glycidol	C004312	decreases^expression	1	1
Hydroquinone	C031927	decreases^expression	1	1
ICG 001	C492448	decreases^expression	1	1
IncobotulinumtoxinA	C545476	decreases^expression	1	1
K 7174	C410337	decreases^expression	1	1
Lactic acid	D019344	decreases^expression	1	1
Leflunomide	D000077339	decreases^expression	1	1
Methyleugenol	C005223	decreases^expression	1	1
Methyl methanesulfonate	D008741	decreases^expression	1	1
Mustard gas	D009151	decreases^expression	1	1
Niclosamide	D009534	decreases^expression	1	1
N-Nitrosopyrrolidine	D009242	decreases^expression	1	1
NSC 689,534	C558013	decreases^expression	1	1
Oxaliplatin	D000077150	decreases^expression	1	1
Palbociclib	C500026	decreases^expression	1	1
Pentabromodiphenyl ether	C086401	decreases^expression	1	1
Perfluorononanoic acid	C584865	decreases^expression	1	1
Phenformin	D010629	decreases^expression	1	1
Phenylmercuric acetate	D010662	decreases^expression	1	1
Potassium dichromate	D011192	decreases^expression	1	1
Quercetin	D011794	decreases^expression	1	1
Rotenone	D012402	decreases^expression	1	1
Soman	D012999	decreases^expression	1	1
Squalestatin 1	C075117	decreases^expression	1	2
Sunitinib	D000077210	decreases^expression	1	1
Systhane	C446685	decreases^expression	1	1
T-2 toxin	D013605	decreases^expression	1	1
Testosterone	D013739	decreases^expression	1	1
Tetrachlorodibenzodioxin	D013749	decreases^expression	2	2
Thapsigargin	D019284	decreases^expression	1	1
Topotecan	D019772	decreases^expression	1	1
Triclosan	D014260	decreases^expression	1	1
Tris(1,3-dichloro-2-propyl)phosphate	C016805	decreases^expression	1	1
Troglitazone	D000077288	decreases^expression	1	1
Tunicamycin	D014415	decreases^expression	2	1
Vincristine	D014750	decreases^expression	1	1

**Table 3:** Potential therapeutic compounds that can result in decreased expression of FAM83F mRNA

Chemical name	Chemical ID	Interaction actions	Reference count	Organism count
2,2’,3’,4,4’,5-hexachlorobiphenyl	C029790	decreases^expression	1	1
2-amino-2-methyl-1-propanol	C006551	decreases^expression	1	1
Dibutyl phthalate	D003993	decreases^expression	1	1
Lenalidomide	D000077269	decreases^expression	1	1
Nickel monoxide	C028007	decreases^expression	1	1
Pomalidomide	C467566	decreases^expression	1	1
Propylthiouracil	D011441	decreases^expression	1	1
Triclosan	D014260	decreases^expression	1	1

**Table 4:** Potential therapeutic compounds that can result in decreased expression of FAM83G mRNA

Chemical name	Chemical ID	Interaction actions	Reference count	Organism count
Acetaminophen	D000082	decreases^expression	1	1
Cisplatin	D002945	decreases^expression	1	1
Dietary fats	D004041	decreases^expression	1	1
Genistein	D019833	decreases^expression	1	1
Progesterone	D011374	decreases^expression	1	1

## Discussion

The *FAM83* family has been recently described as a novel oncogene family [12, 13], but there was limited research about their function in cancer development. Notably, their function in breast cancer has not been addressed. All eight *FAM83* family members had a highly conserved N-terminal DUF1669 domain. This domain, with unknown function, was necessary to drive the oncogenic transformation [5, 7, 8, 26]. Therefore, FAM83 proteins might be potential therapeutic targets for cancer treatment [26]. Our report evaluated the mRNA expression, prognostic effect, and regulation mechanism of the *FAM83* family proteins in breast malignancies for the first time.

In this study, we found that FAM83A, FAM83D, FAM83F, and FAM83G were higher expressed in breast cancer tissues compared to normal tissues and had an adverse effect on the patients’ survival outcomes. However, other members of the *FAM83* family did not have such effects. We performed a functional and pathway enrichment analysis of genes co-expressed with FAM83A, FAM83D, FAM83F, and FAM83G. We found that these genes played an essential role in several cancer-related biological processes such as cell proliferation, G2/M transition of the mitotic cell cycle, and regulation of apoptosis. Additionally, they were enriched in different cancer-related signaling pathways, including the Hippo, Hedgehog, and PI3K/AKT pathways. We also found that the expression of FAM83A, FAM83D, FAM83F, and FAM83G mRNA was regulated by promoter DNA methylation in most CpG islands and significantly related to immune cell infiltration. At last, we screened different compounds using the CTD database and identify potential therapeutic compounds which can decrease the mRNA expression of FAM83A, FAM83D, FAM83F, and FAM83G mRNA for breast cancer. These cancer-related signaling pathways, the results of promoter methylation and the potential compounds for FAM83A, FAM83D, FAM83F, and FAM83G were found by us for the first time.

It has been previously reported that FAM83A expression was elevated in breast cancer, pancreatic cancer, and hepatocellular cancer [27–29]. Cuiping Liu et al. also found that miR-613 in breast cancer negatively regulated FAM83A expression. Moreover, miR-613-induced FAM83A decreased expression can impair triple-negative breast cancer stemness and tumorigenesis in vitro and in vivo [27]. Sun-Young Lee et al. found that FAM83A can interact with and cause phosphorylation of c-RAF and PI3K p85, upstream of MAPK and downstream of EGFR, to confer resistance to EGFR-TKIs in breast cancer [13]. Moreover, forced FAM83A expression can enhance breast cancer cell proliferation and invasion and make it resistant to TKIs. Contradictorily, decreased FAM83A expression had the reverse effect [30]. For HER2 + breast cancer, Courtney A. Bartel, and Mark W. Jackson found that decreased FAM83A expression can inhibit HER2 + breast cancer cell proliferation, promote cell apoptosis, and inhibit the PI3K pathway; however, this was not related to trastuzumab sensitivity [31]. For the role of FAM83A in other tumors, it was reported that FAM83A can activate TGFβ and Wnt/β-catenin pathway to promote the proliferation of cancer stem cells and the progress of pancreatic cancer [32]. Richtmann, S et al. also found FAM83A can serve as prognostic biomarker and new potential new therapeutic targets for non-small cell lung cancer [33]. Our results similarly follow the above studies. We found that FAM83A was highly expressed in breast cancer tissues and had an adverse effect on the patient’s survival. And our RT-PCR results also validated this.

Many studies have found that FAM83D can promote the development of colorectal cancer [34], invasive ovarian cancer [35], non-small-cell lung cancer [36, 37], and hepatocellular cancer [38, 39]. For instance, FAM83D can promote the proliferation of hepatocellular can by activating MAPK pathway [38, 39] or inhibiting the activation of tumor suppressor gene FBXW7 [40]. Walian et al. also demonstrated that overexpression of FAM83D in MCF10A normal breast cells could promote breast cell proliferation, invasion, migration, and finally, malignant transformation [41]. Similarly, Xiuming Zhai et al. found that the expression level of FAM83D was related to the RFS of triple-negative breast cancer patients and could serve as a novel biomarker for breast cancer diagnosis [42]. Our results were similar with those reported by Xiuming Zhai et al., as FAM83D mRNA high expression was associated with a poor survival outcome in overall breast cancer patients. Moreover, Xiuming Zhai et al. validated the high expression of FAM83D in triple-negative breast cancer tissues by RT-PCR [42]. And our RT-PCR results validated this in overall breast cancer tissues too.

Few reports are available for FAM83F and FAM83G. Gongchun Fan et al. found that FAM83F was upregulated in lung adenocarcinoma cells, and the high expression was related to cancer progression and poor survival. Also, FAM83F can reduce the sensitivity to cisplatin or docetaxel of lung adenocarcinoma cells [43]. Mohammed Salama et al. also found that forced FAM83F expression can activate mutant forms of p53 and enhance cell migration [44]. The expression of FAM83F was significantly higher in esophageal squamous cell carcinoma (ESCC) than in normal tissues. FAM83F was the downstream target of miR-143. Under the action of miR-143, the decreased expression of FAM83F can inhibit proliferation, invasion, and migration of ESCC cells, and induce G1/G0 arrest in ESCC cells [45]. We did not find the results about FAM83F in breast cancer. We found that FAM83F was highly expressed in breast cancer and was significantly related to poor survival outcomes and validated the significantly high expression in breast cancer by RT-PCR.

Only a few studies found that FAM83G can control Wnt signaling by association with casein kinase 1α [46]. FAM83G can also regulate cytoskeletal dynamics and organization as loss of FAM83G can cause severe defects in F-actin organization and distribution and lamellipodial organization, resulting in impaired cell migration [47]. For the role of FAM83G in cancer, present studies only found that the expression of FAM83G was higher in hepatocellular cancer tissues than in normal tissues. And the high expression of FAM83G was associated with early metastasis and high recurrence rate of hepatocellular cancer. FAM83G can serve as a poor prognostic factor for patients with hepatocellular carcinoma. Furthermore, both in vivo and in vitro experiments confirmed that overexpression of FAM83G significantly promoted the proliferation, migration and invasion of hepatocellular carcinoma cells, whereas inhibition of its expression reversed the above results. Mechanistic analysis showed that FAM83G overexpression was accompanied by over-activation of PI3K/AKT pathway signaling, increased expression of Cyclin D1 and decreased expression of p21, and increased expression of epithelial-mesenchymal transition-related factors. FAM83G can also activate PI3K/AKT signaling by directly binding to the PI3K-p85 subunit and promote its phosphorylation [48]. FAM83G was also found as a novel inducer of apoptosis [49]. In contrast, we identified FAM83G as an oncogene, and our database results showed that it might inhibit apoptosis. This result needs to be validated by in vitro experiments in the future.

We would like to acknowledge the limitations of our study. First, we did not validate the prognostic effect of FAM83A, FAM83D, FAM83F, and FAM83G in breast cancer clinical samples. Second, we need to perform a functional experiment to explore the role of FAM83A, FAM83D, FAM83F, and FAM83G in the development of breast cancer in vitro and in vivo. At last, we would also like to explore the regulatory mechanism of FAM83A, FAM83D, FAM83F, and FAM83G in breast cancer in the future.

## Conclusions

In conclusion, we found that FAM83A, FAM83D, FAM83F, and FAM83G were highly expressed in breast cancer tissues and had an adverse effect on breast cancer patients’ survival outcomes. Genes co-expressed with FAM83A, FAM83D, FAM83F, and FAM83G might be enriched in the Hippo, Hedgehog, and PI3K/AKT signaling pathways, therefore playing an essential role in the development and progression of breast cancer. FAM83A, FAM83D, FAM83F, and FAM83G may serve as potential therapeutic targets for breast cancer clinical treatment, and further research needs to be done in this direction.

## Supplementary Information

**Additional file 1: Figure S1.** The expression of FAM83A based on different clinicopathological characteristics by UALCAN database.

**Additional file 2: Figure S2.** The expression of FAM83D based on different clinicopathological characteristics by UALCAN database.

**Additional file 3: Figure S3.** The expression of FAM83F based on different clinicopathological characteristics by UALCAN database.

**Additional file 4: Figure S4.** The expression of FAM83G based on different clinicopathological characteristics by UALCAN database.

**Additional file 5: Figure S5.** Exploration the expression and subcellular localization of FAM83A by the HPA database.

**Additional file 6: Figure S6.** Exploration the expression and subcellular localization of FAM83D by the HPA database.

**Additional file 7: Figure S7.** Exploration the expression and subcellular localization of FAM83F by the HPA database.

**Additional file 8: Figure S8.** Exploration the expression and subcellular localization of FAM83G by the HPA database.

**Additional file 9: Figure S9.** The relationships between immune cell infiltration and the expression of FAM83A, FAM83D, FAM83F and FAM83G by “ESTIMATE” analysis tool.

**Additional file 10: Table S1.** The sequence of primers.

**Additional file 11: Table S2.** The basic clinicopathological characteristics of these twenty patients.

## Data Availability

These data and materials can be available from corresponding authors for rational reasons.

## Abbreviations

FAM83: FAMily with sequence similarity 83
RFS: Relapse-free survival
HR: Hazard ratios
OS: Overall survival
HPA: Human Protein Atlas
BRCA: Breast invasive carcinoma
CTD: Comparative Toxicogenomics Database
TNBC: Triple negative breast cancer
ESCC: Esophageal squamous cell carcinoma
