# Supplementary figures and images for: Comprehensive analysis of the expression, prognostic significance, and function of FAM83 family members in breast cancer
Source: World J Surg Oncol. 2022 Jun 1;20:172. doi: 10.1186/s12957-022-02636-9 (PMC9158143; doi:10.1186/s12957-022-02636-9)

# Supp Fig 1

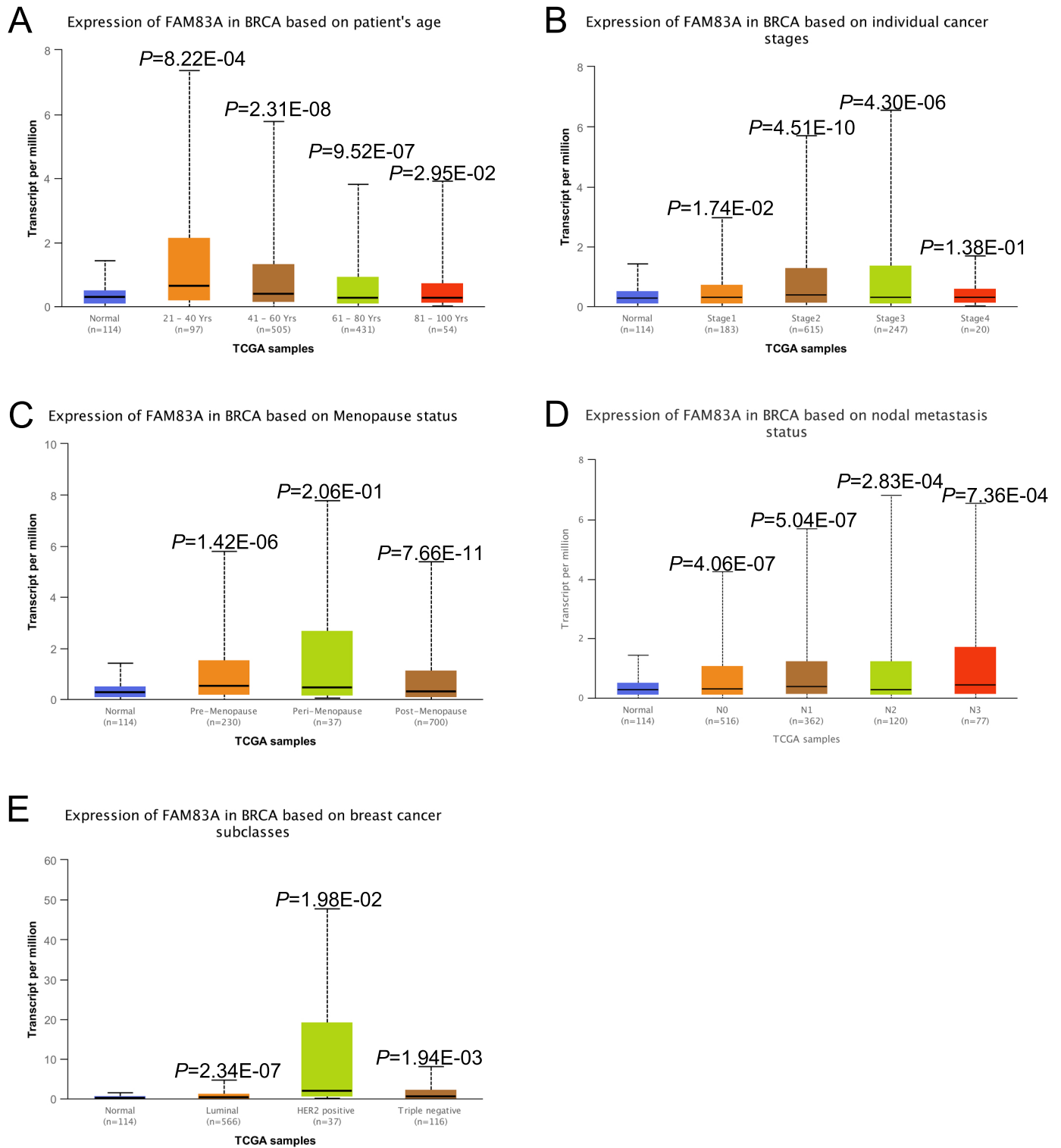

Supplement: Supplementary file 1 — Additional file 1: Figure S1. The expression of FAM83A based on different clinicopathological characteristics by UALCAN database. [file 12957_2022_2636_MOESM1_ESM.pdf]

Supp Fig 2

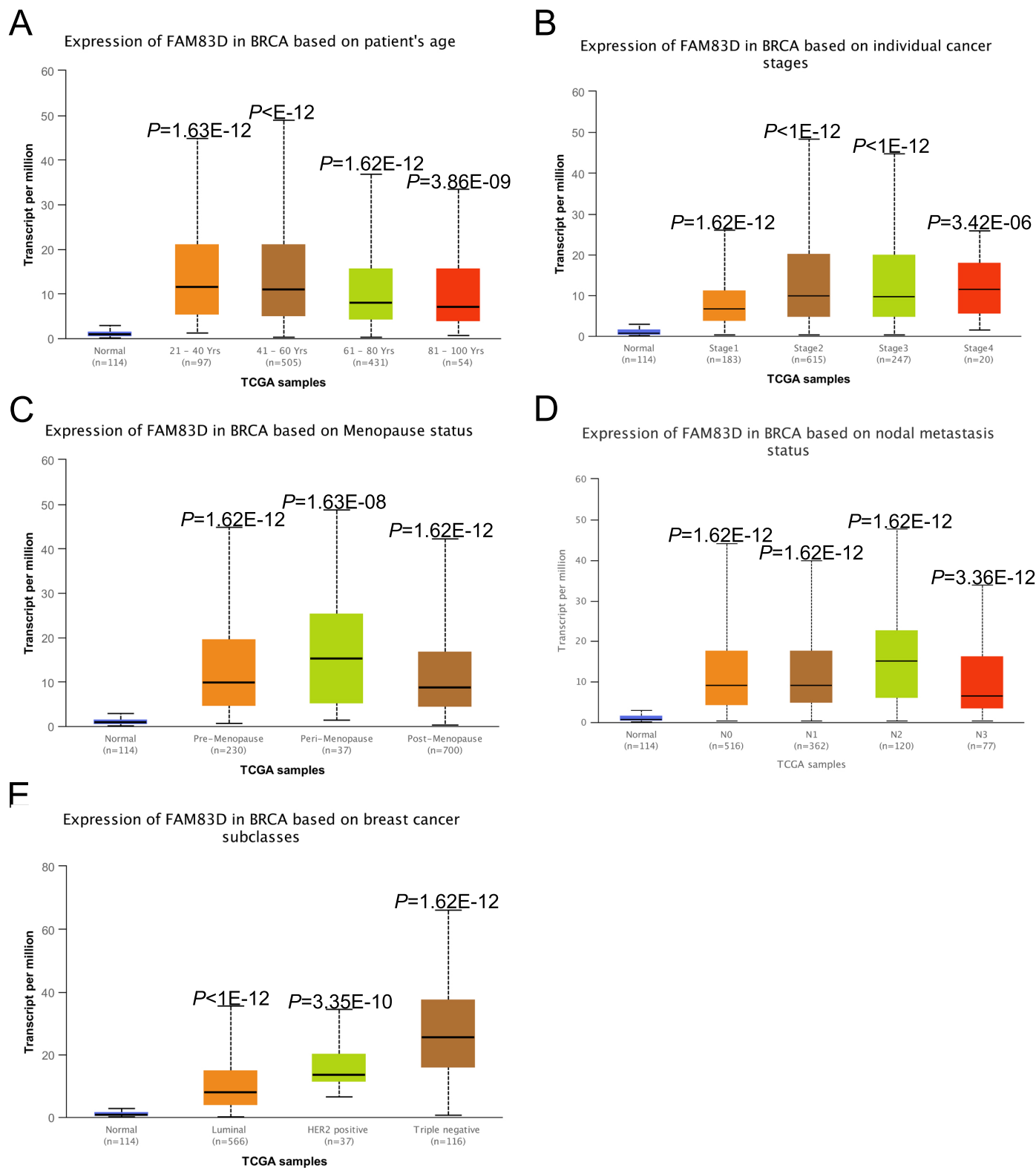

Supplement: Supplementary file 2 — Additional file 2: Figure S2. The expression of FAM83D based on different clinicopathological characteristics by UALCAN database. [file 12957_2022_2636_MOESM2_ESM.pdf]

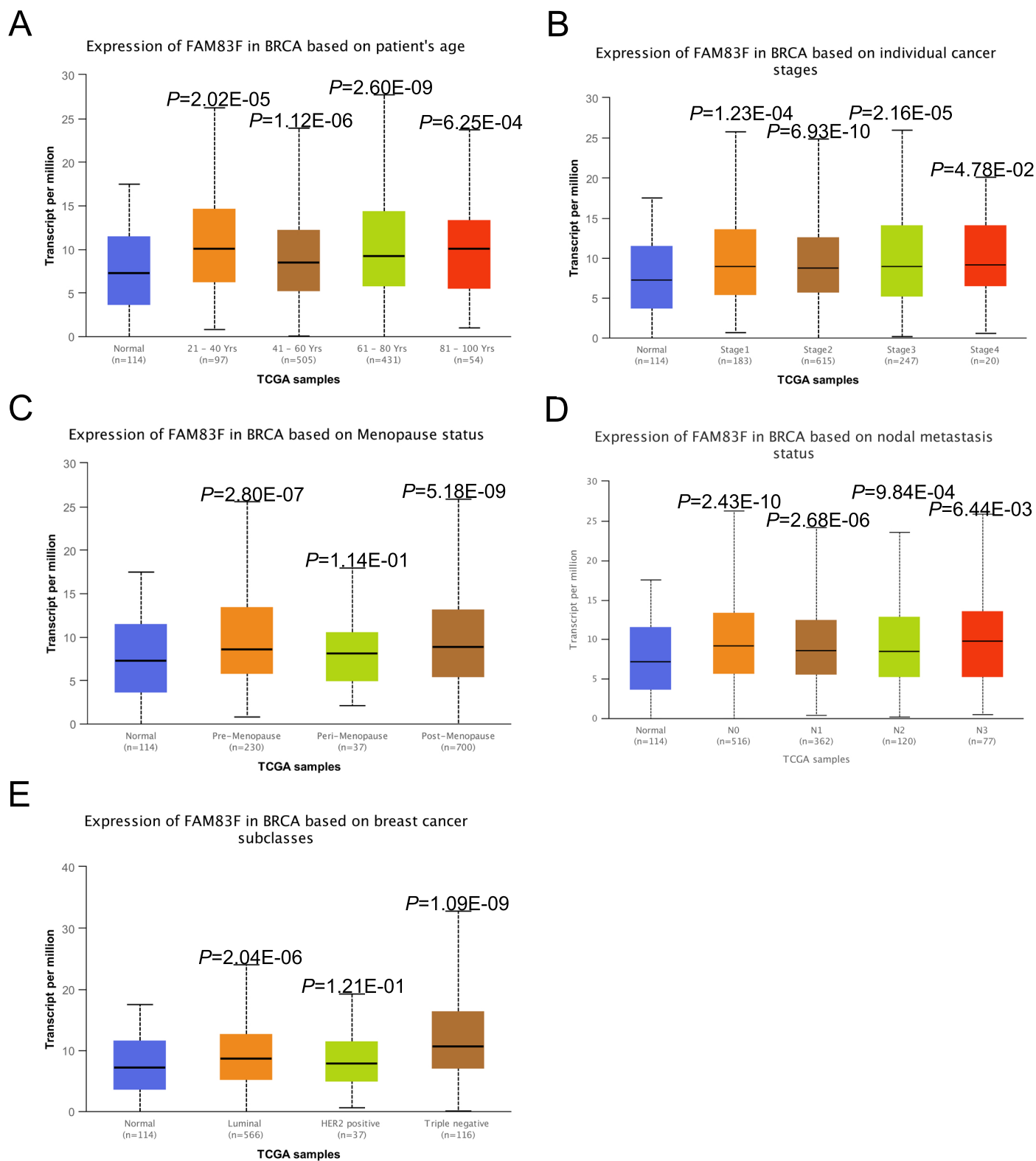

Supplement: Supplementary file 3 — Additional file 3: Figure S3. The expression of FAM83F based on different clinicopathological characteristics by UALCAN database. [file 12957_2022_2636_MOESM3_ESM.pdf]

# Supp Fig 4

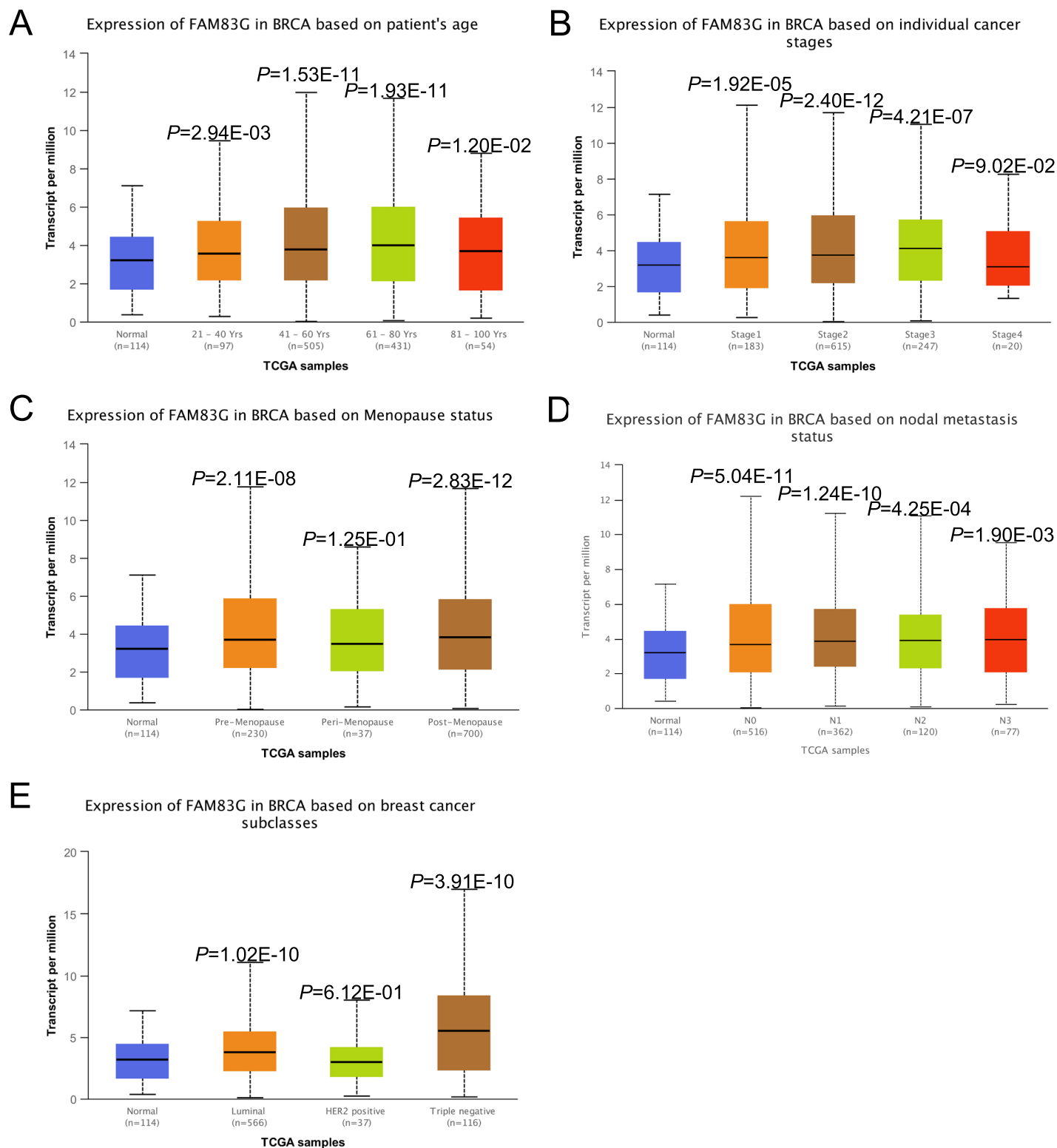

Supplement: Supplementary file 4 — Additional file 4: Figure S4. The expression of FAM83G based on different clinicopathological characteristics by UALCAN database. [file 12957_2022_2636_MOESM4_ESM.pdf]

## Supp Fig 5

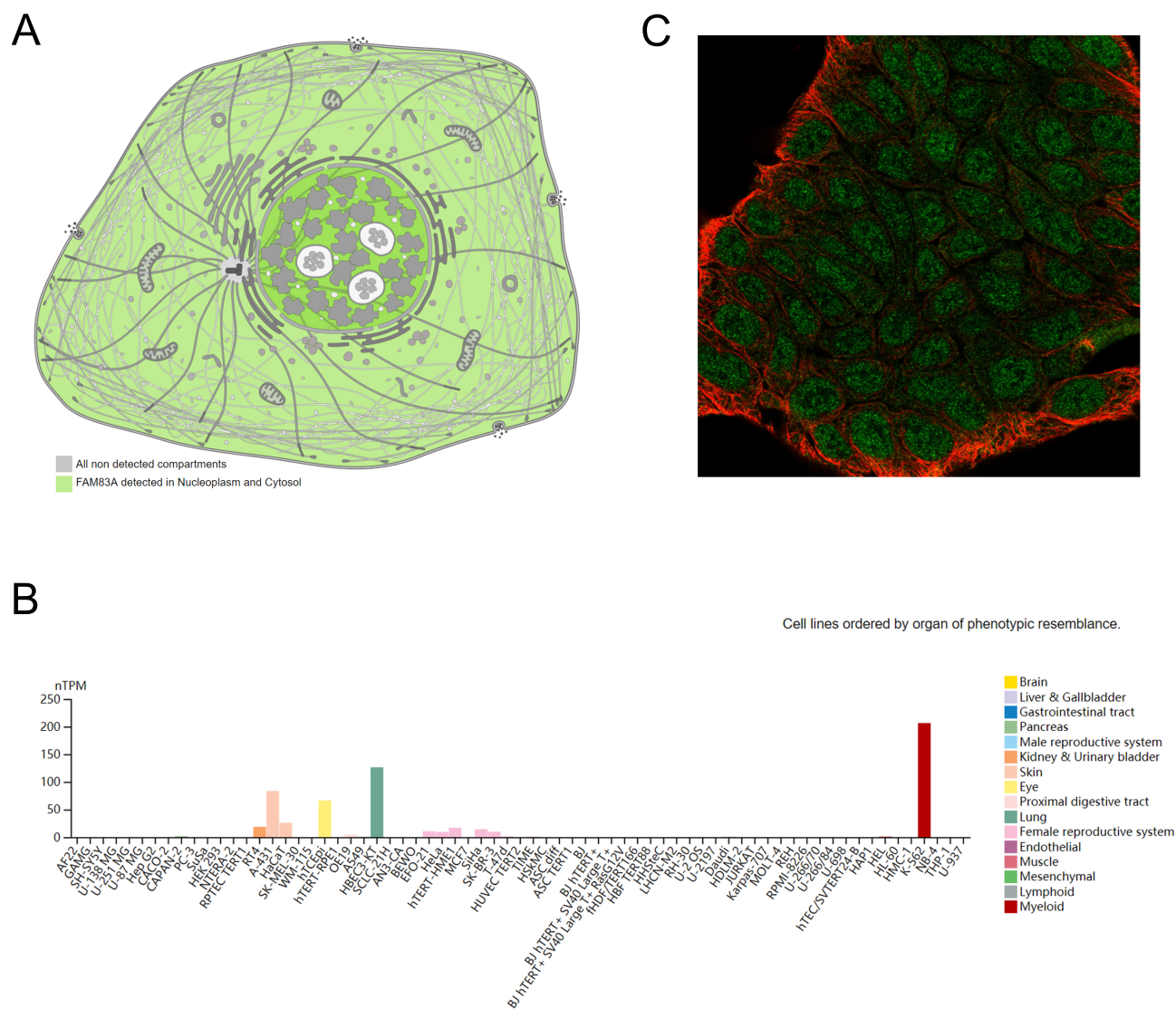

Supplement: Supplementary file 5 — Additional file 5: Figure S5. Exploration the expression and subcellular localization of FAM83A by the HPA database. [file 12957_2022_2636_MOESM5_ESM.pdf]

## Supp Fig 6

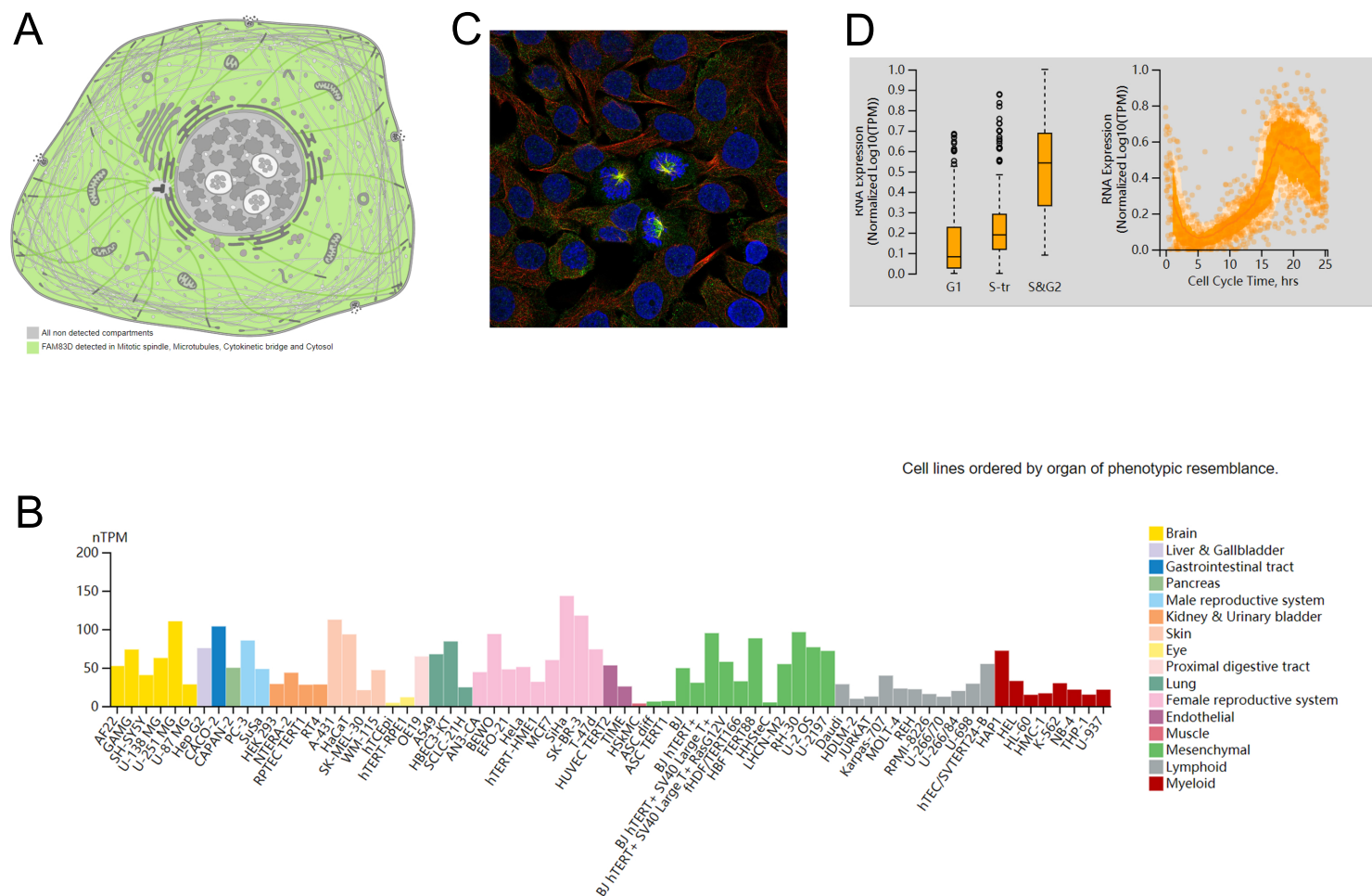

Supplement: Supplementary file 6 — Additional file 6: Figure S6. Exploration the expression and subcellular localization of FAM83D by the HPA database. [file 12957_2022_2636_MOESM6_ESM.pdf]

Supp Fig 7

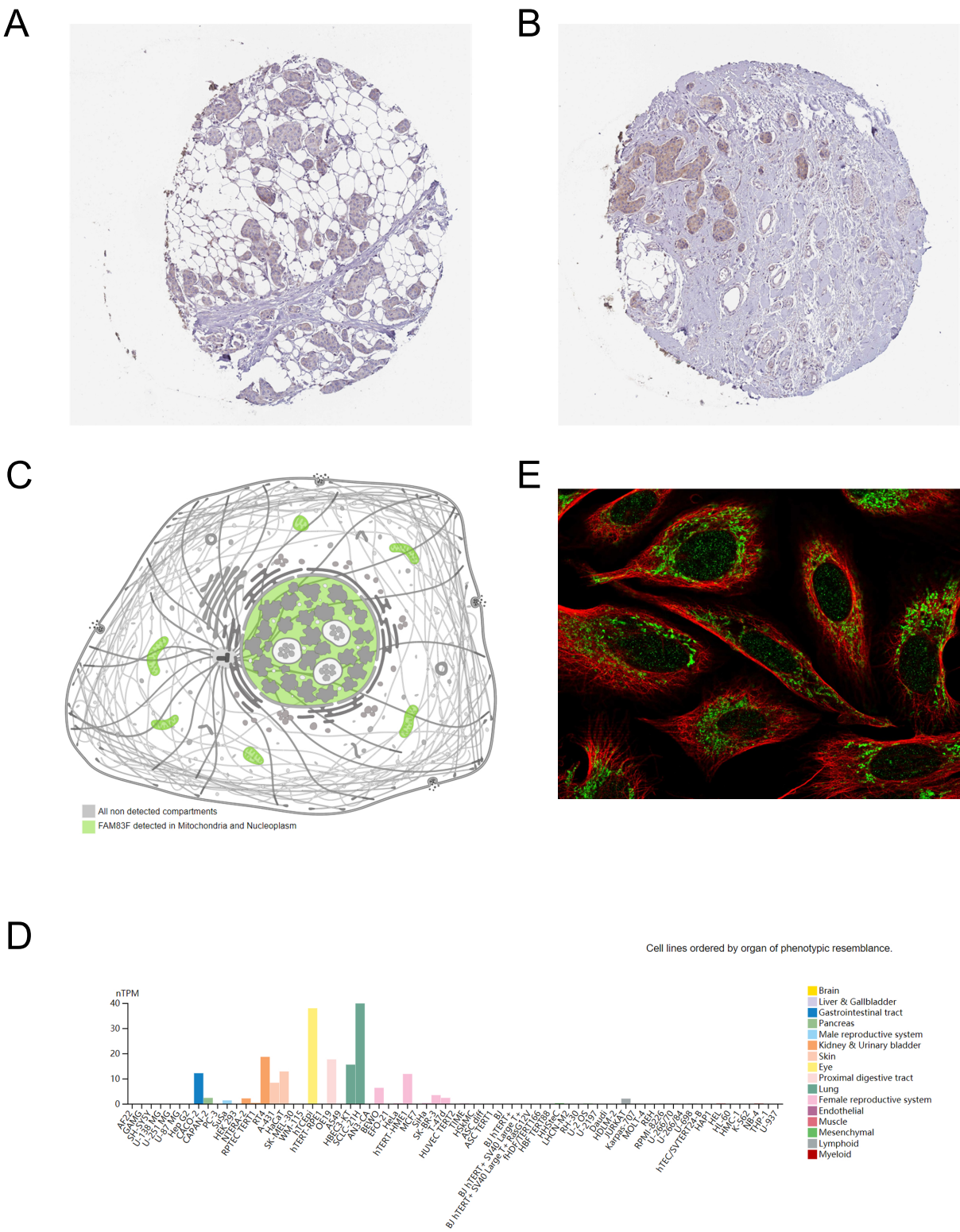

Supplement: Supplementary file 7 — Additional file 7: Figure S7. Exploration the expression and subcellular localization of FAM83F by the HPA database. [file 12957_2022_2636_MOESM7_ESM.pdf]

Supp Fig 8

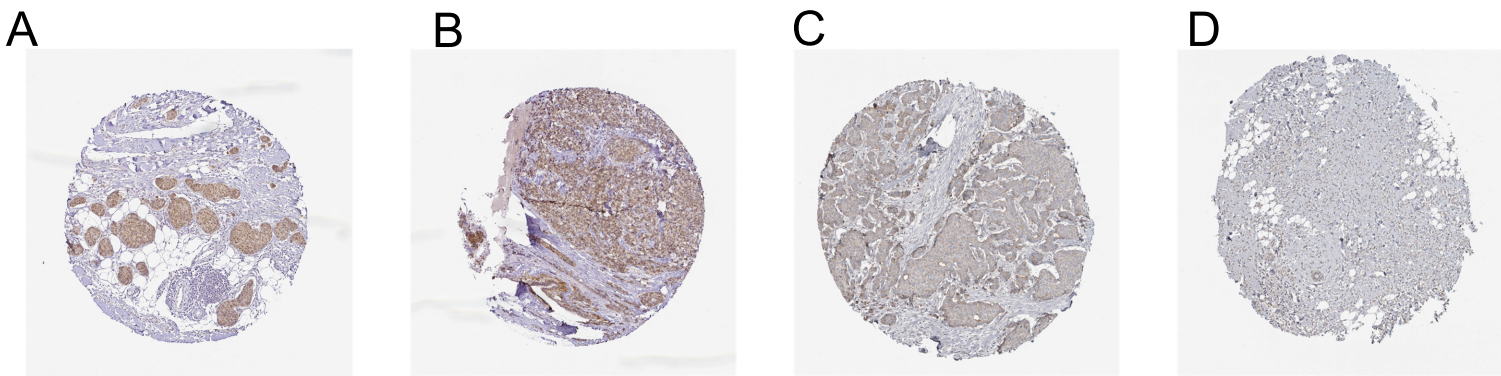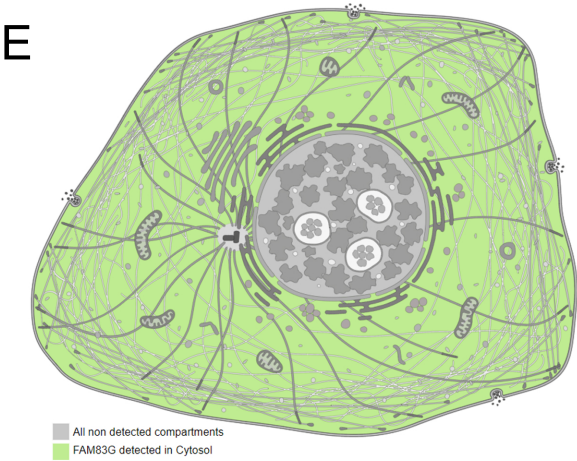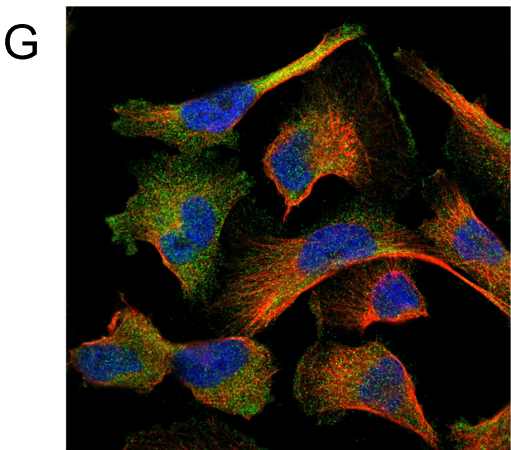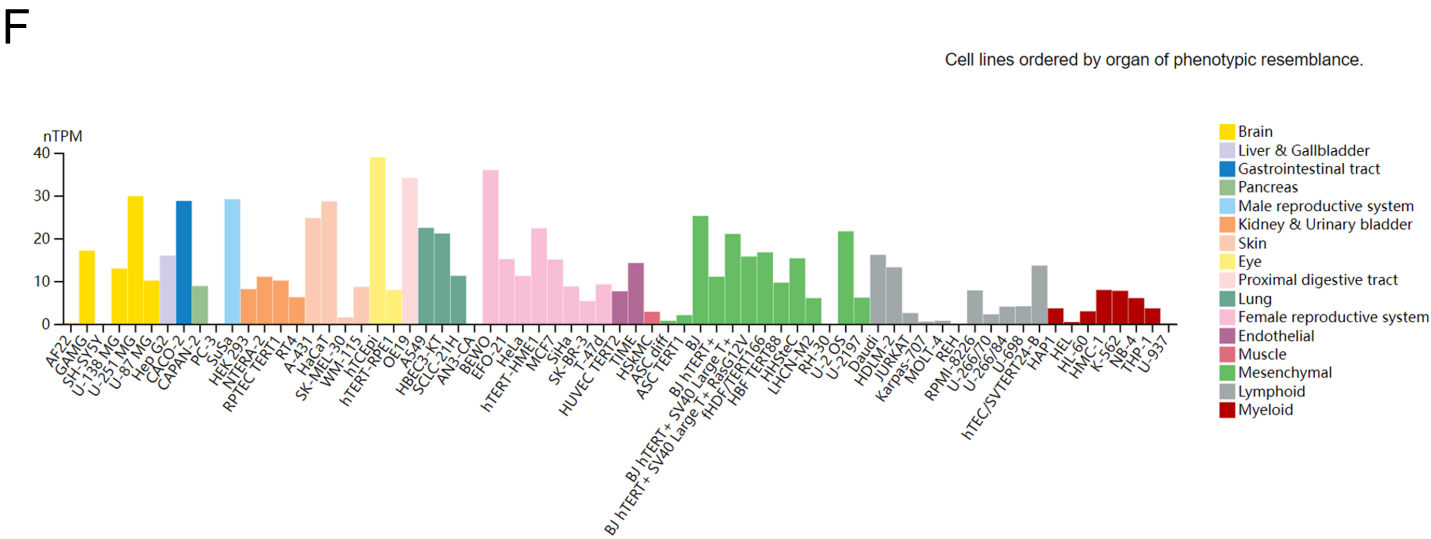

Supplement: Supplementary file 8 — Additional file 8: Figure S8. Exploration the expression and subcellular localization of FAM83G by the HPA database. [file 12957_2022_2636_MOESM8_ESM.pdf]

# Supp Fig 9

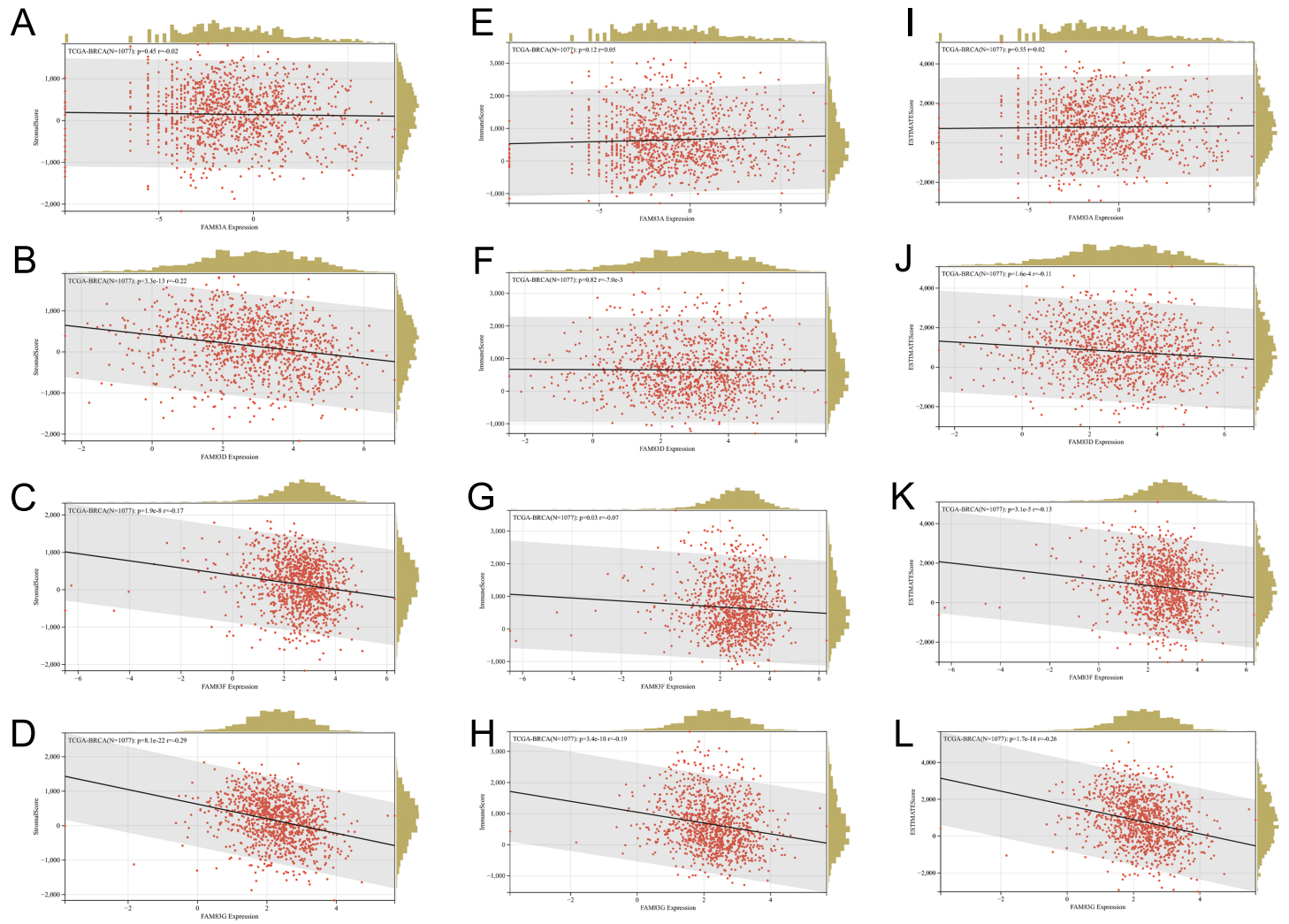

Supplement: Supplementary file 9 — Additional file 9: Figure S9. The relationships between immune cell infiltration and the expression of FAM83A, FAM83D, FAM83F and FAM83G by “ESTIMATE” analysis tool. [file 12957_2022_2636_MOESM9_ESM.pdf]
