# Supplementary material for: Comprehensive analysis of the expression, prognostic significance, and function of FAM83 family members in breast cancer
Source: World J Surg Oncol. 2022 Jun 1;20:172. doi: 10.1186/s12957-022-02636-9 (PMC9158143; doi:10.1186/s12957-022-02636-9)
Supplement: Supplementary file 10 — Additional file 10: Table S1. The sequence of primers. [file 12957_2022_2636_MOESM10_ESM.docx]

Supplementary table 1 The sequence of primers

| Gene name | Forward | Reverse |
| --- | --- | --- |
| FAM83A | 5’-TCAAGCACAACAACATCAGAGACCTC-3’ | 5’-CTCCACATCCGTGAACACATCCATC-3’ |
| FAM83B | 5’-TTGGAGGAGGATGAGGAGGAAGTTAC-3’ | 5’-TCACATCAAGTAAAGCAGCAATGGAAAC-3’ |
| FAM83C | 5’-GGAGGTGGTGATTGCAGTGATACG-3’ | 5’-GTTAGGGTCTGACAGTTGGCGATG-3’ |
| FAM83D | 5’-GGCTCTCCTCTCTCAATTTCTGGATATG-3’ | 5’-TCCTGTGATAGTCCGAACTGTCATTAAC-3’ |
| FAM83E | 5’-ATTGTTGACGCCTTCAGCCTTGAG-3’ | 5’-CCACCTATGACCGAGGGTTTCTGG-3’ |
| FAM83F | 5’-GCTTCCTGAAAGACCTGGTTACGG-3’ | 5’-AGAGACCATCCTGCCATCCTTCTG-3’ |
| FAM83G | 5’-ATCAAAGAGGTGGTGCGGAAGATG-3’ | 5’-GTCCAGCAGGTCCTTGAAGATGTC-3’ |
| FAM83H | 5’-ACCAGGTGCTCCATAATGAGTCAAAAG-3’ | 5’-CCCTTCTGCTCGATAAATCCTGTAGTTG-3’ |
| GAPDH | 5’-CCTTCCGTGTCCCCACT-3’ | 5’-GCCTGCTTCACCACCTTC-3’ |
