# Supplementary material for: Comprehensive analysis of the expression, prognostic significance, and function of FAM83 family members in breast cancer
Source: World J Surg Oncol. 2022 Jun 1;20:172. doi: 10.1186/s12957-022-02636-9 (PMC9158143; doi:10.1186/s12957-022-02636-9)
Supplement: Supplementary file 11 — Additional file 11: Table S2. The basic clinicopathological characteristics of these twenty patients. [file 12957_2022_2636_MOESM11_ESM.docx]

Supplementary table 2 The basic clinicopathological characteristics of these twenty patients

| Clinicopathological characteristics | Patients 1 | Patients 2 | Patients 3 | Patients 4 | Patients 5 | Patients 6 | Patients 7 | Patients 8 | Patients 9 | Patients 10 |
| --- | --- | --- | --- | --- | --- | --- | --- | --- | --- | --- |
| Gender | Female | Female | Female | Female | Female | Female | Female | Female | Female | Female |
| Age | 64 | 50 | 68 | 59 | 44 | 58 | 50 | 38 | 50 | 67 |
| T grade | 1 | 2 | 2 | 1 | 1 | 1 | 2 | 2 | 1 | 1 |
| N grade | 0 | 0 | 2 | 1 | 1 | 1 | 0 | 0 | 0 | 0 |
| Menopause status | Postmenopausal | Premenopausal | Postmenopausal | Postmenopausal | Premenopausal | Postmenopausal | Postmenopausal | Premenopausal | Postmenopausal | Postmenopausal |
| Subclasses | Luminal A | Luminal A | Luminal A | Luminal A | Luminal A | Luminal B | Luminal B | Luminal B | Luminal B | Luminal B |
| Metastasis | No | No | No | No | No | No | No | No | No | No |
|  | Patients 11 | Patients 12 | Patients 13 | Patients 14 | Patients 15 | Patients 16 | Patients 17 | Patients 18 | Patients 19 | Patients 20 |
| Gender | Female | Female | Female | Female | Female | Female | Female | Female | Female | Female |
| Age | 53 | 39 | 55 | 66 | 43 | 47 | 31 | 46 | 43 | 56 |
| T grade | 1 | 2 | 2 | 2 | 2 | 2 | 2 | 2 | 2 | 2 |
| N grade | 3 | 3 | 0 | 0 | 0 | 3 | 0 | 0 | 0 | 2 |
| Menopause status | Postmenopausal | Premenopausal | Postmenopausal | Postmenopausal | Premenopausal | Premenopausal | Premenopausal | Premenopausal | Premenopausal | Postmenopausal |
| Subclasses | HER2 positive | HER2 positive | HER2 positive | HER2 positive | HER2 positive | TNBC | TNBC | TNBC | TNBC | TNBC |
| Metastasis | No | No | No | No | No | No | No | No | No | No |
